# Estimation in regret-regression using quadratic inference functions with ridge estimator

**DOI:** 10.1371/journal.pone.0271542

**Published:** 2022-07-21

**Authors:** Nur Raihan Abdul Jalil, Nur Anisah Mohamed, Rossita Mohamad Yunus

**Affiliations:** Institute of Mathematical Sciences, Faculty of Science, Universiti Malaya, Kuala Lumpur, Malaysia; University of Sargodha, PAKISTAN

## Abstract

In this paper, we propose a new estimation method in estimating optimal dynamic treatment regimes. The quadratic inference functions in myopic regret-regression (QIF-MRr) can be used to estimate the parameters of the mean response at each visit, conditional on previous states and actions. Singularity issues may arise during computation when estimating the parameters in ODTR using QIF-MRr due to multicollinearity. Hence, the ridge penalty was introduced in rQIF-MRr to tackle the issues. A simulation study and an application to anticoagulation dataset were conducted to investigate the model’s performance in parameter estimation. The results show that estimations using rQIF-MRr are more efficient than the QIF-MRr.

## Introduction

A dynamic treatment regime (DTR) is a branch of personalized medicine that uses information from the patient to minimize health problems. In reality, the treatment response for each patient is different, which influenced to the development of the DTR and personalized medicine. The advantages of personalized medicine include the following: a cutback in the total cost of health care; the patient receiving an option to intensive health care by deciding an optimal decision for the treatment; and increased compliance and devotion towards treatment [1].

Throughout the years, researchers have shown an interest in DTR. For example, [2] described anticoagulant data to obtain an optimal strategy for the warfarin-treated patient who is at risk of thrombosis (i.e., abnormal blood clotting). Another example of DTR is the estimation of the optimal time by [3] for an asymptomatic HIV-infected subject to start highly active retroviral therapy (HAART). Others include [4–7].

DTR, also known as adaptive strategies, adaptive interventions, or treatment policies, is a multi-stage decision rule of personalized medicine. It defines a set of decision rules to determine the treatment of an individual based on their health condition and treatment history. The term “dynamic” indicates a variation of treatment using the patient’s current state and previous treatment decisions. The purpose of DTR is to optimize the mean outcome for each patient, also called optimal dynamic treatment regime (ODTR).

[8] proposed a regret function to estimate ODTR semi-parametrically and parameterized the optimal rules using the iterative minimization of regrets (IMOR) method. Following the development of ODTR, [9] proposed a regret-regression method where the ODTR was estimated by incorporating the regret function into regression modeling. A doubly robust version of the regret regression by [10] claimed to be equivalent to a reduced form of the efficient g-estimation method [11, 12].

[13] introduced the myopic regret-regression (MRr) which is a short-term strategy of the regret-regression. In a short-term strategy, the ODTR was estimated at each time point. The MRr provides good estimates when the outcome is measured through time. However, in the previous works mentioned above, no attention has been given to the correlation within-subject. ODTR is actually a longitudinal dataset where the observations of one subject are dependent on each other over time. Many studies made use of longitudinal data, for example in [14–16].

To estimate ODTR for correlated data, [13] proposed a method called QIF-MRr which integrates the MRr into the quadratic inference functions (QIF). The QIF method was proposed by [17] where it is an extension to the generalized estimating equations (GEE) [18] which has widely been used in the analysis of longitudinal and correlated data [19–22]. Hence, the QIF-MRr is able to provide unbiased and efficient estimates even with the misspecified working correlation structure.

In the early years [23], proposed the penalized estimating equations, which considered a bridge penalty, and applied them to the GEE method. Meanwhile, [24] proposed the penalized QIF by using the penalized spline and QIF method. In this paper, we proposed an estimation strategy called the ridge quadratic inference function for myopic regret-regression (rQIF-MRr) to estimate ODTR for correlated data. The ridge penalty, also known as *L*_2_ penalty [25]. The reason we choose the ridge penalty over the other penalties is that the ridge penalty can control inflation and general instability related to the least square estimates [26] and it also can solve singularity issues [27].

The paper is organized as follows: We first define the notations and assumptions needed throughout the paper. Then, we propose the rQIF-MRr in estimating ODTR. The proposed method is illustrated using simulation and application to anticoagulant dataset.

## Methods

In this section, we will propose a ridge quadratic inference function for myopic regret-regression (rQIF-MRr) to estimate the parameter for ODTR. We follow the notations obtained from [9]. Suppose,

- *j* = 1, 2, …, *K* is the number of time visits, with *K* is the final time visit for subject *i*.
- *n* is the sample size where subject *i* = 1, 2, …, *n*.
- *S*_*j*_ represents the current state of the subject *i* at visit *j*.
- *A*_*j*_ represents an action or treatment decision made for subject *i* at visit *j*.
-
${\bar{S}}_{j}=\left(S_{1},S_{2},\ldots ,S_{j}\right)$ is the cumulative information of the states for subject *i* measured from the first to the *j*th visit.
-
${\bar{A}}_{j}=\left(A_{1},A_{2},\ldots ,A_{j}\right)$ is the cumulative actions given to subject *i* from the first to the *j*th visit based on the previous history $\left({\bar{S}}_{j-1},{\bar{A}}_{j-1}\right)$.
- The action given at visit *j* is determined by the state of the subject at visit *j* with the actions given at the previous visits, *A*_*j*−1_.
- The states and actions $\left({\bar{S}}_{j},{\bar{A}}_{j}\right)$ were assumed to be independent between the subjects and dependent within a subject.
- The response *Y*_*j*_ were measured at each visit *j*.
- From the notion of the potential outcome or counterfactual [28, 29], *a*_*j*_ is a set of all possible actions that can be given at visit *j*.
-
${\bar{S}}_{j}\left({\bar{a}}_{j-1}\right)=\left(S_{1},S_{2}\left(a_{1}\right),\ldots ,S_{j}\left({\bar{a}}_{j-1}\right)\right)$ is the potential state history under the possible action ${\bar{a}}_{j-1}$.
-
$Y({\bar{a}}_{K})$ is the potential outcome under the possible action ${\bar{a}}_{K}$.
-
$d_{j}^{opt}$ is the optimal dynamic treatment regime which optimizes the expected value of outcome *Y*_*j*_.
-
$E(Y\left({\underline{d}}_{j}^{opt}\right)|{\bar{S}}_{j},{\bar{A}}_{j-1})$ is the expected value of the potential outcome or counterfactual final responses
-
$E(Y\left(a_{j},{\underline{d}}_{j+1}^{opt}\right)|{\bar{S}}_{j},{\bar{A}}_{j-1})$ is the expected value of the potential outcome if action *a*_*j*_ is chosen at time *j* and then subsequently the optimal decision rules are followed.

Three assumptions were made in this study, which are consistency, no unmeasured confounders, and positivity. The assumption of consistency is when the observed outcome *Y* is equal to the potential outcome $Y({\bar{a}}_{K})$ and the observed state history ${\bar{S}}_{K}$ is equal to the potential state history ${\bar{S}}_{{\bar{a}}_{K-1}}$ under the observed treatment *a*_*K*_ = *A*_*K*_. The treatment is given in a way that it is possible for all the treatment options to be assigned to all patients in the population under consideration.

The assumption of no unmeasured confounders is that the decision for each treatment depends only on the observed states and treatment history. For any regime ${\bar{a}}_{K}$, the action given at visit *j*, *A*_*j*_ is independent of any future or potential states or outcome given the previous history, for *j* = 1, 2, …, *K*. If there is no drop-out, the assumption is equivalent to the exchangeability. If the subjects are censored, further assumption is needed where the censoring is non-informative conditional on history. That is, the potential outcome of a censored patient will follow the same distribution as the uncensored patients.

The third assumption about positivity is that the optimal treatment regime has a nonzero or positive probability of being observed in the data. In continuous treatment, the optimal treatment regime is identifiable from the observed data. The assumption may be theoretically and practically violated. A theoretical violation occurs when the study’s design prevents a patient from receiving a specific therapy. Practical violations, on the other hand, occur when a portion of the patient has a very low probability of receiving therapy.

### Ridge quadratic inference function for myopic regret-regression (rQIF-MRr)

Suppose the mean response of the MRr from [13] is

$$\begin{array}{cc} h_{j} & =E(Y_{j}|{\bar{S}}_{j},{\bar{A}}_{j})\\ & =\beta_{0}+\phi_{j}\left({\bar{S}}_{j}|{\bar{S}}_{j-1},{\bar{A}}_{j-1};\beta \right)-\mu_{j}\left(A_{j}|{\bar{S}}_{j},{\bar{A}}_{j-1},\psi \right), \end{array}$$ (1)

where $\phi_{j}\left({\bar{S}}_{j}|{\bar{S}}_{j-1},{\bar{A}}_{j-1};\beta \right)=\beta_{j}^{T}\left({\bar{S}}_{j-1},{\bar{A}}_{j-1}\right)Z_{j}$. The coefficients, $\beta_{j}^{T}\left({\bar{S}}_{j-1},{\bar{A}}_{j-1}\right)$ depend on the history before visit *j* of the states and actions [9, 10]. Meanwhile, the residuals, $Z_{j}=S_{j}-E\left(S_{j}|{\bar{S}}_{j-1},{\bar{A}}_{j-1}\right)$ is a linear combination between *S*_*j*_ and the expected value of *S*_*j*_ given the history, $\left({\bar{S}}_{j-1},{\bar{A}}_{j-1}\right)$. The regret function $\mu_{j}\left(A_{j}|{\bar{S}}_{j},{\bar{A}}_{j-1},\psi \right)$ is zero if the optimal action is selected at visit *j*. Otherwise, the regret function is positive since the aim is to maximize the response *Y*_*j*_ [9].

[17] defines *R*(*ρ*) to be the working correlation matrix with parameter *ρ*, and the inverse function of *R*^−1^(*ρ*) can be approximated by a linear combination of several basis matrices defined as

$$\begin{array}{c} R^{-1}\left(\rho \right)=\sum_{l=1}^{m}\tau_{l}M_{l}, \end{array}$$ (2)

where *τ*_1_ are unknown coefficients and *M*_*l*_ are known basis matrices.

There are several types of working correlation structures [17], but in this paper, we only focus on three common types of working correlation structures, which are the first order autoregressive, AR(1), exchangeable and unspecified working correlation structures. The estimating equation of the QIF-MRr is

$$\begin{array}{c} \sum_{i=1}^{n}{\left(\frac{\partial h_{i}}{\partial \left(\beta ,\psi \right)}\right)}^{T}D_{i}^{-\frac{1}{2}}\left(\tau_{1}M_{1}+\ldots +\tau_{m}M_{m}\right)D_{i}^{-\frac{1}{2}}\left(Y_{i}-h_{i}\right), \end{array}$$ (3)

and can be written in the form of its extended score as

$$\begin{array}{c} g_{n}\left(\beta ,\psi \right)=\frac{1}{n}\sum_{i=1}^{n}g_{i}\left(\beta ,\psi \right)=\frac{1}{n}(\begin{array}{c} \sum_{i=1}^{n}{\left(\frac{\partial h_{i}}{\partial \left(\beta ,\psi \right)}\right)}^{T}D_{i}^{-\frac{1}{2}}M_{1}D_{i}^{-\frac{1}{2}}\left(Y_{i}-h_{i}\right)\\ \sum_{i=1}^{n}{\left(\frac{\partial h_{i}}{\partial \left(\beta ,\psi \right)}\right)}^{T}D_{i}^{-\frac{1}{2}}M_{2}D_{i}^{-\frac{1}{2}}\left(Y_{i}-h_{i}\right)\\ \vdots \\ \sum_{i=1}^{n}{\left(\frac{\partial h_{i}}{\partial \left(\beta ,\psi \right)}\right)}^{T}D_{i}^{-\frac{1}{2}}M_{m}D_{i}^{-\frac{1}{2}}\left(Y_{i}-h_{i}\right) \end{array}) \end{array}$$ (4)

where *g*_*i*_(*β*, *ψ*) is the estimating equations from Eq (3), and the linear coefficients, *τ*_*l*_ can be viewed as nuisance parameters [24]. The *D*_*i*_ is a diagonal matrix of the marginal variances for subject *i*, and *M*_1_, …, *M*_*m*_ are the basis functions for the working correlation matrix with dimension (*K* × *K*). The derivatives of the *h*_*j*_(*β*, *ψ*) term, ∂*h*^*T*^/∂(*β*, *ψ*) and vector *g*_*i*_((*β*, *ψ*)) have (*p* × *K*) dimension where *p* is the number of parameters (*β*, *ψ*).

As there are more equations than the parameters, the generalized method of moments (GMM) from [30], can be applied to create the QIF-MRr as

$$\begin{array}{c} Q_{n}\left(\beta ,\psi \right)=g_{n}^{T}C_{n}^{-1}g_{n}, \end{array}$$ (5)

where,

$$\begin{array}{c} C_{n}={\frac{1}{n}}^{2}\sum_{i=1}^{n}g_{i}^{T}\left(\beta ,\psi \right))g_{i}\left(\beta ,\psi \right). \end{array}$$

The parameters *β* and *ψ* can be estimated by setting the extended score, *g*_*n*_(*β*, *ψ*) in Eq (4) as close to zero as possible, which is by minimizing the *Q*_*n*_(*β*, *ψ*) function as

$$\begin{array}{c} \left(\hat{\beta },\hat{\psi }\right)=\text{arg}\;\underset{\left(\beta ,\psi \right)}{\text{min}}g_{n}^{T}C_{n}^{-1}g_{n}. \end{array}$$ (6)

Singularity problem often occurs during estimation using QIF-MRr in ODTR. [27] used a ridge-regression to solve singularity problem. Thus, by applying the penalized QIF from [31], we introduce the ridge penalty in QIF-MRr and define a new *Q*_*n*_ function of Eq (5) as

$$\begin{array}{c} rQ_{n}\left(\beta ,\psi \right)=g_{n}^{T}C_{n}^{-1}g_{n}+\lambda \sum_{v=1}^{p}{\left|{\left(\beta ,\psi \right)}_{v}\right|}^{2};\lambda \geq 0 \end{array}$$ (7)

where λ is the tuning parameter, and $\sum_{v=1}^{p}{\left|{\left(\beta ,\psi \right)}_{v}\right|}^{2}$ is a ridge penalty function where *v* = 1, …, *p* is the number of parameter (*β*, *ψ*), and *p* is the total number of parameter (*β*, *ψ*). The penalty function will act as a weight during estimation, and stabilize the estimation of the parameter in the computation. The rQIF-MRr minimizes the parameters *β* and *ψ* as

$$\begin{array}{c} \left(\hat{\beta },\hat{\psi }\right)=\text{arg}\;\underset{\left(\beta ,\psi \right)}{\text{min}}g_{n}^{T}C_{n}^{-1}g_{n}+\lambda \sum_{v=1}^{p}{\left|{\left(\beta ,\psi \right)}_{v}\right|}^{2} \end{array}$$ (8)

## Results and discussions

The parameter estimates of the proposed method were investigated using simulations. The simulation dataset were generated sing the scenario from [8] in order to estimate the mean response *Y*_*j*_ at each visit. Let *i* = 1, 2, …, *n* be the observed subject, and *n* is the sample size. Then, *j* = 1, 2, …, *K* is the time visit where *K* = 10 is the final visit.

We generate the first state *S*_1_ for each *i* from a normal distribution with mean 0.5 and variance 0.01. For the second state onwards, the states *S*_*j*_ ∼ *N*(*m*_*j*_, 0.01), where

$$\begin{array}{c} m_{j}=0.5+0.2S_{j-1}-0.07A_{j-1}, \end{array}$$

for *j* = 2, 3, …, *K*. In this simulation, we only consider one action per visit. The action, *A*_*j*_ were generated from uniform distribution, *A*_*j*_ ∼ *U*{0, 1, 2, 3}.

The regret function is defined as

$$\begin{array}{c} \mu_{j}\left(a_{j}|{\bar{S}}_{j},{\bar{A}}_{j-1};\psi \right)=\psi_{1}\left|a_{j}-\psi_{2}-\psi_{3}S_{j}\right| \end{array}$$ (9)

where $\underset{\mu_{j}}{\text{min}}\mu_{j}\left(a_{j}|{\bar{S}}_{j},{\bar{A}}_{j-1};\psi \right)=0$. We define

$$\begin{array}{c} I_{j}=\{\begin{array}{ccc} 1 & \text{if} & A_{j}-\psi_{2}-\psi_{3}S_{j}\geq 0\\ -1 & \text{if} & A_{j}-\psi_{2}-\psi_{3}S_{j}<0 \end{array} \end{array}$$ (10)

where *I*_*j*_ is a vector of length *K* for each subject.

The response, *Y*_*j*_ is generated from a normal distribution with mean,

$$\begin{array}{cc} h_{j}\left(\beta ,\psi \right) & =E(Y_{j}|{\bar{S}}_{j},{\bar{A}}_{j})\\ & =\beta_{0}+\beta_{1}Z_{j}-\psi_{1}\left|a_{j}-\psi_{2}-\psi_{3}S_{j}\right| \end{array}$$ (11)

and variance $\sigma_{Y}^{2}=0.64$, where

$$\begin{array}{cc} Z_{j} & =S_{j}-E\left(S_{j}\right)\\ & =S_{j}-m_{j}. \end{array}$$

The residuals, $\in ∼N(0,\sigma_{Y}^{2}\Sigma )$ where Σ is obtained from an autoregressive true correlation matrix. Applying the Cholesky decomposition, Σ = *CC*^*T*^ which decomposed a positive-definite matrix into the product of a lower triangular matrix and its conjugate transpose. We take **ϵ** = *C* **W** where **W** ∼ *N*(0, *I*_*R*_). Thus,

$$\begin{array}{cc} var(\epsilon ) & =\sigma_{Y}^{2}Cvar\left(W\right)C^{T}\\ & =\sigma_{Y}^{2}CC^{T}\\ & =\sigma_{Y}^{2}\Sigma \end{array}$$

Then, the response variable for each subject *i* is

$$\begin{array}{c} Y_{j}=\beta_{0}+\beta_{1}Z_{j}-\mu_{j}\left(A_{j}|{\bar{S}}_{j},{\bar{A}}_{j-1};\psi \right)+\epsilon . \end{array}$$

The correlation in the response variable was levelled into three levels: *ρ* = 0.1, 0.5, 0.95. *ρ* = 0.1 indicates a low correlation, while *ρ* = 0.5 is a medium correlation, and *ρ* = 0.95 is a high correlation. The initial parameter values for the coefficients are *β* = {3, −5} and *ψ* = {1.5, 0.1, 5.5}.

To estimate the parameters, we first regress each *S*_*j*_ on history $\left({\bar{S}}_{j-1},{\bar{A}}_{j-1}\right)$ and define *Z*_*j*_ [9]. Then, we differentiate *h*_*j*_(*β*, *ψ*) from Eq (11) with respect to *β*_0_, *β*_1_, *ψ*_1_, *ψ*_2_ and *ψ*_3_ to obtain ∂*h*/∂(*β*, *ψ*). For each subject *i*, the partial derivative with respect to *β*_0_ is

$$\begin{array}{c} \frac{\partial h}{\partial \beta_{0}}=(\begin{array}{c} 1\\ \vdots \\ 1 \end{array}) \end{array}$$

and the partial derivative with respect to *β*_1_ is

$$\begin{array}{c} \frac{\partial h}{\partial \beta_{1}}=Z \end{array}$$

where *Z* is the residual vector for subject *i*. The partial derivatives with respect to *ψ*_1_, *ψ*_2_ and *ψ*_3_ are then

$$\begin{array}{c} \frac{\partial h}{\partial \psi_{1}}=-I, \end{array}$$

$$\begin{array}{c} \frac{\partial h}{\partial \psi_{2}}=\psi_{1}I, \end{array}$$

$$\begin{array}{c} \frac{\partial h}{\partial \psi_{3}}=\psi_{1}S_{j}I. \end{array}$$

where *I* is an indicators sign of the regrets for subject *i* from Eq (10).

The parameters $\left(\hat{\beta },\hat{\psi }\right)$ can be obtained using the *optim* built-in function in R by minimizing Eq (8). The tuning parameter, λ was obtained using a cross-validation technique [32]. The optimal tuning parameter for this simulation is λ = 0.01. For the simulation, we used bootstrap resampling 1000 times with two sample sizes (*n* = 25 and *n* = 500). [33] considered that *n* = 25 as a small sample size. Hence, we used a sample size *n* = 25 to compare the performance in estimates between the rQIf-MRr and QIF-MRr methods. We fit the models (i.e. QIF-MRr and rQIF-MRr) using AR(1), exchangeable, and unspecified working correlation structures.

For the AR(1) working correlation structure, the inverse working correlation structure can be written as

$$\begin{array}{c} R^{-1}\left(\rho \right)=\tau_{0}M_{0}+\tau_{1}M_{1}+\tau_{2}M_{2} \end{array}$$

where *M*_0_ is an identity matrix

$$\begin{array}{c} M_{0}=(\begin{array}{cccc} 1 & 0 & \ldots & 0\\ 0 & 1 & \ldots & 0\\ \vdots & \vdots & \ddots & \vdots \\ 0 & 0 & \ldots & 1 \end{array}), \end{array}$$

*M*_1_ is a matrix with 1 on the two main off-diagonals and 0 elsewhere,

$$\begin{array}{c} M_{1}=(\begin{array}{cccccc} 0 & 1 & & & \ldots & 0\\ 1 & 0 & 1 & & \ldots & 0\\ 0 & 1 & 0 & 1 & \ldots & 0\\ \vdots & \vdots & \ddots & \ddots & \ddots & \vdots \\ 0 & \ldots & \ldots & \ldots & \ldots & 0 \end{array}) \end{array}$$

and *M*_2_ is a matrix with 1 on the corners (1, 1) and (*K*, *K*) and 0 elsewhere

$$\begin{array}{c} M_{2}=(\begin{array}{cccc} 1 & 0 & \ldots & 0\\ 0 & 0 & \ldots & 0\\ \vdots & \vdots & \ddots & \vdots \\ 0 & 0 & \ldots & 1 \end{array}). \end{array}$$

Here *τ*_0_ = (1 + *ρ*^2^)/(1 − *ρ*^2^), *τ*_1_ = (−*ρ*)/(1 − *ρ*^2^) and *τ*_2_ = (−*ρ*^2^)/(1 − *ρ*^2^).

For an exchangeable working correlation structure, *R*(*ρ*) consists of 1’s on the diagonal and *ρ*’s everywhere off-diagonal. Then, *R*^−1^ is given as

$$\begin{array}{c} R^{-1}\left(\rho \right)=\tau_{0}M_{0}+\tau_{1}M_{1} \end{array}$$

where *M*_0_ is an identity matrix

$$\begin{array}{c} M_{0}=(\begin{array}{cccc} 1 & 0 & \ldots & 0\\ 0 & 1 & \ldots & 0\\ \vdots & \vdots & \ddots & \vdots \\ 0 & 0 & \ldots & 1 \end{array}) \end{array}$$

and *M*_1_ is a matrix with diagonal elements 0 and off-diagonal elements 1

$$\begin{array}{c} M_{1}=(\begin{array}{cccc} 0 & 1 & \ldots & 1\\ 1 & 0 & \ldots & 1\\ \vdots & \vdots & \ddots & \vdots \\ 1 & 1 & \ldots & 0 \end{array}). \end{array}$$

Note that, *τ*_0_ = −{(*K* − 2)*ρ* + 1}/{(*K* − 1)*ρ*^2^ − (*K* − 2)*ρ* − 1} and *τ*_1_ = *ρ*/{(*K* − 1)*ρ*^2^ − (*K* − 2)*ρ* − 1} and *K* is the dimension of *R*.

The unspecified working correlation structure can be used to determine the working correlation structure when there is some difficulty and challenges in obtaining it. For the unspecified working correlation structure, the basis matrices *M*_0_ = *I*_*n*_ and $M_{1}=\hat{U}$, where

$$\begin{array}{c} \hat{U}=\frac{1}{N}\Sigma \left(Y_{i}-h_{i}\right){\left(Y_{i}-h_{i}\right)}^{T}, \end{array}$$ (12)

and the matrix $\hat{U}$ is a consistent estimator of the variance matrix of *Y* [19].

The correctly specified working correlation structure is when we use the AR(1) working correlation structure to generate the data and fit the models. Otherwise, the model is considered a misspecified working correlation structure. The results below give the mean value of the parameter estimates for 1000 bootstrap resampling (Mean), standard error (SE), and the root mean square error (RMSE).

With sample sizes of *n* = 25 and *n* = 500 at different correlation values, *ρ*, Table 1 compares parameter estimations using QIF-MRr and rQIF-MRr. The rQIF-MRr is more efficient than the QIF-MRr for small and large sample sizes, with small SE and RMSE.

**Table 1:** Parameter estimation for correctly specified working correlation structure of the QIF-MRr and rQIF-MRr with different correlation values, *ρ*.

*ρ*	Methods	Coefficients	*n* = 25			*n* = 500		
			Mean	SE	RMSE	Mean	SE	RMSE
*ρ* = 0.1	QIF-MRr	*β*_0_	3.0442	0.3685	0.3712	3.0596	0.3803	0.3850
		*β*_1_	-4.9017	0.1962	0.2195	-4.8988	0.2135	0.2362
		*ψ*_1_	1.5558	0.3601	0.3644	1.5561	0.3894	0.3934
		*ψ*_2_	0.1956	0.2691	0.2856	0.2037	0.2665	0.2859
		*ψ*_3_	5.6546	0.2147	0.2646	5.6269	0.2171	0.2515
	rQIF-MRr	*β*_0_	3.0528	0.2974	0.3020	3.0324	0.3120	0.3137
		*β*_1_	-4.9154	0.1731	0.1926	-4.9166	0.1656	0.1854
		*ψ*_1_	1.5316	0.3404	0.3419	1.5333	0.3342	0.3358
		*ψ*_2_	0.2168	0.2530	0.2786	0.2400	0.2625	0.2975
		*ψ*_3_	5.6035	0.1928	0.2188	5.6036	0.1999	0.2252
*ρ* = 0.5	QIF-MRr	*β*_0_	3.0311	0.3521	0.3535	3.0292	0.3428	0.3441
		*β*_1_	-4.9223	0.1916	0.2067	-4.9212	0.1961	0.2113
		*ψ*_1_	1.6086	0.3687	0.3844	1.5549	0.3340	0.3384
		*ψ*_2_	0.2245	0.3203	0.3436	0.2323	0.2759	0.3060
		*ψ*_3_	5.6301	0.2163	0.2524	5.6416	0.2295	0.2697
	rQIF-MRr	*β*_0_	3.0352	0.3101	0.3121	3.0448	0.2970	0.3003
		*β*_1_	-4.9165	0.1789	0.1974	-4.9266	0.1720	0.1870
		*ψ*_1_	1.5513	0.3242	0.3282	1.5363	0.3446	0.3465
		*ψ*_2_	0.2155	0.2656	0.2896	0.2428	0.2522	0.2899
		*ψ*_3_	5.6063	0.2031	0.2292	5.6026	0.1939	0.2194
*ρ* = 0.95	QIF-MRr	*β*_0_	3.0824	0.3606	0.3699	3.0514	0.3118	0.3160
		*β*_1_	-4.8987	0.2140	0.2367	-4.9067	0.2045	0.2248
		*ψ*_1_	1.5349	0.3998	0.4013	1.5637	0.3731	0.3785
		*ψ*_2_	0.2027	0.2925	0.3100	0.2107	0.2674	0.2894
		*ψ*_3_	5.6263	0.2216	0.2551	5.6302	0.2201	0.2557
	rQIF-MRr	*β*_0_	3.0503	0.2901	0.2944	3.0339	0.3014	0.3033
		*β*_1_	-4.9251	0.1698	0.1856	-4.9098	0.1679	0.1907
		*ψ*_1_	1.5355	0.3352	0.3371	1.5332	0.3613	0.3628
		*ψ*_2_	0.2267	0.2618	0.2908	0.2281	0.2720	0.3007
		*ψ*_3_	5.6058	0.1846	0.2127	5.6060	0.1979	0.2245

The results for the correctly specified working correlation structure was shown in Table 1, where the data is generated and fitted using AR(1) working correlation structure. The advantage of using the QIF-MRr and rQIF-MRr in estimation is that the estimates are still efficient even with the misspecified working correlation structure.

The estimation for the misspecified working correlation structures for both QIF-MRr and rQIF-MRr are given in Tables 2–4. For low correlation, *ρ* = 0.1 in Table 2, the parameter estimation for rQIF-MRr are unbiased and efficient with small SE and RMSE even with misspecified working correlation structures. The correctly specified AR(1) for rQIF-MRr gives slightly smaller SE compared to exchangeable and unspecified working correlation structures.

**Table 2:** Parameter estimation of the misspecification working correlation structures for the QIF-MRr and rQIF-MRr at low correlation value, *ρ* = 0.1.

*ρ*	Methods	Correlation Structure	Coefficients	*n* = 25			*n* = 500		
				Mean	SE	RMSE	Mean	SE	RMSE
*ρ* = 0.1	QIF-MRr	AR(1)	*β*_0_	3.0442	0.3685	0.3712	3.0596	0.3803	0.3850
			*β*_1_	-4.9017	0.1962	0.2195	-4.8988	0.2135	0.2362
			*ψ*_1_	1.5558	0.3601	0.3644	1.5561	0.3894	0.3934
			*ψ*_2_	0.1956	0.2691	0.2856	0.2037	0.2665	0.2859
			*ψ*_3_	5.6546	0.2147	0.2646	5.6269	0.2171	0.2515
		Exchangeable	*β*_0_	3.0645	0.3672	0.3728	3.0790	0.3613	0.3699
			*β*_1_	-4.8821	0.2071	0.2383	-4.8961	0.2096	0.2340
			*ψ*_1_	1.5157	0.4074	0.4077	1.5514	0.3795	0.3830
			*ψ*_2_	0.1980	0.2941	0.3100	0.1909	0.2613	0.2767
			*ψ*_3_	5.6276	0.1952	0.2332	5.6270	0.2277	0.2607
		Unspecified	*β*_0_	2.9806	0.4761	0.4765	2.9593	0.4881	0.4898
			*β*_1_	-4.9094	0.2694	0.2843	-4.9037	0.2389	0.2576
			*ψ*_1_	1.5900	0.5022	0.5103	1.5900	0.5650	0.5721
			*ψ*_2_	0.2376	0.3304	0.3579	0.2361	0.2575	0.2913
			*ψ*_3_	5.6526	0.2461	0.2896	5.6581	0.2357	0.2838
	rQIF-MRr	AR(1)	*β*_0_	3.0528	0.2974	0.3020	3.0324	0.3120	0.3137
			*β*_1_	-4.9154	0.1731	0.1926	-4.9166	0.1656	0.1854
			*ψ*_1_	1.5316	0.3404	0.3419	1.5333	0.3342	0.3358
			*ψ*_2_	0.2168	0.2530	0.2786	0.2400	0.2625	0.2975
			*ψ*_3_	5.6035	0.1928	0.2188	5.6036	0.1999	0.2252
		Exchangeable	*β*_0_	3.0552	0.3411	0.3455	3.0373	0.3554	0.3573
			*β*_1_	-4.9108	0.1659	0.1883	-4.9051	0.1643	0.1897
			*ψ*_1_	1.5284	0.3452	0.3464	1.5199	0.3650	0.3656
			*ψ*_2_	0.2122	0.2545	0.2781	0.1989	0.2618	0.2799
			*ψ*_3_	5.5959	0.2063	0.2275	5.6178	0.1911	0.2245
		Unspecified	*β*_0_	3.0111	0.4069	0.4071	2.9980	0.3949	0.3949
			*β*_1_	-4.9116	0.1951	0.2142	-4.9123	0.1928	0.2119
			*ψ*_1_	1.5272	0.4894	0.4902	1.5415	0.5086	0.5103
			*ψ*_2_	0.2339	0.2960	0.3249	0.2326	0.2512	0.2840
			*ψ*_3_	5.6149	0.2122	0.2413	5.6203	0.1876	0.2228

**Table 3:** Parameter estimation of the misspecification working correlation structures for the QIF-MRr and rQIF-MRr at medium correlation value, *ρ* = 0.5.

*ρ*	Methods	Correlation Structure	Coefficients	*n* = 25			*n* = 500		
				Mean	SE	RMSE	Mean	SE	RMSE
*ρ* = 0.5	QIF-MRr	AR(1)	*β*_0_	3.0311	0.3521	0.3535	3.0292	0.3428	0.3441
			*β*_1_	-4.9223	0.1916	0.2067	-4.9212	0.1961	0.2113
			*ψ*_1_	1.6086	0.3687	0.3844	1.5549	0.3340	0.3384
			*ψ*_2_	0.2245	0.3203	0.3436	0.2323	0.2759	0.3060
			*ψ*_3_	5.6301	0.2163	0.2524	5.6416	0.2295	0.2697
		Exchangeable	*β*_0_	3.0382	0.4092	0.4110	3.0596	0.4076	0.4119
			*β*_1_	-4.8863	0.1930	0.2240	-4.8863	0.1998	0.2298
			*ψ*_1_	1.5769	0.3343	0.3430	1.5301	0.3806	0.3818
			*ψ*_2_	0.1984	0.2691	0.2865	0.2049	0.2762	0.2954
			*ψ*_3_	5.6305	0.2191	0.2550	5.6194	0.1988	0.2319
		Unspecified	*β*_0_	3.0109	0.4180	0.4181	3.0342	0.4024	0.4038
			*β*_1_	-4.9027	0.2338	0.2533	-4.9305	0.2197	0.2304
			*ψ*_1_	1.5958	0.4666	0.4764	1.6050	0.5100	0.5207
			*ψ*_2_	0.2211	0.3208	0.3429	0.2631	0.2868	0.3300
			*ψ*_3_	5.6165	0.2055	0.2363	5.5904	0.2032	0.2224
	rQIF-MRr	AR(1)	*β*_0_	3.0352	0.3101	0.3121	3.0448	0.2970	0.3003
			*β*_1_	-4.9165	0.1789	0.1974	-4.9266	0.1720	0.1870
			*ψ*_1_	1.5513	0.3242	0.3282	1.5363	0.3446	0.3465
			*ψ*_2_	0.2155	0.2656	0.2896	0.2428	0.2522	0.2899
			*ψ*_3_	5.6063	0.2031	0.2292	5.6026	0.1939	0.2194
		Exchangeable	*β*_0_	3.0594	0.3355	0.3407	3.0422	0.3355	0.3382
			*β*_1_	-4.9105	0.1606	0.1838	-4.9146	0.1771	0.1966
			*ψ*_1_	1.5171	0.3443	0.3447	1.5107	0.3758	0.3759
			*ψ*_2_	0.2007	0.2496	0.2691	0.2083	0.2561	0.2781
			*ψ*_3_	5.6083	0.1878	0.2168	5.6162	0.1998	0.2311
		Unspecified	*β*_0_	3.0182	0.4152	0.4156	2.9904	0.4173	0.4174
			*β*_1_	-4.9158	0.1944	0.2118	-4.9178	0.1868	0.2041
			*ψ*_1_	1.5059	0.4972	0.4972	1.5257	0.5301	0.5307
			*ψ*_2_	0.2308	0.2858	0.3143	0.2471	0.2620	0.3005
			*ψ*_3_	5.6149	0.2098	0.2392	5.6232	0.1913	0.2275

**Table 4:** Parameter estimation of the misspecification working correlation structures for the QIF-MRr and rQIF-MRr at high correlation value, *ρ* = 0.95.

*ρ*	Methods	Correlation Structure	Coefficients	*n* = 25			*n* = 500		
				Mean	SE	RMSE	Mean	SE	RMSE
*ρ* = 0.95	QIF-MRr	AR(1)	*β*_0_	3.0824	0.3606	0.3699	3.0514	0.3118	0.3160
			*β*_1_	-4.8987	0.2140	0.2367	-4.9067	0.2045	0.2248
			*ψ*_1_	1.5349	0.3998	0.4013	1.5637	0.3731	0.3785
			*ψ*_2_	0.2027	0.2925	0.3100	0.2107	0.2674	0.2894
			*ψ*_3_	5.6263	0.2216	0.2551	5.6302	0.2201	0.2557
		Exchangeable	*β*_0_	3.0917	0.3967	0.4071	3.0433	0.3566	0.3593
			*β*_1_	-4.9110	0.2219	0.2391	-4.8956	0.1836	0.2112
			*ψ*_1_	1.5109	0.3888	0.3890	1.5925	0.3413	0.3536
			*ψ*_2_	0.2113	0.2789	0.3003	0.2072	0.2833	0.3030
			*ψ*_3_	5.6337	0.2271	0.2635	5.6022	0.1977	0.2226
		Unspecified	*β*_0_	3.0447	0.4625	0.4646	3.0129	0.4968	0.4970
			*β*_1_	-4.8935	0.1912	0.2189	-4.9421	0.2372	0.2442
			*ψ*_1_	1.5760	0.4772	0.4832	1.5813	0.4911	0.4978
			*ψ*_2_	0.2113	0.2811	0.3023	0.2701	0.3556	0.3941
			*ψ*_3_	5.6389	0.2353	0.2732	5.6423	0.2583	0.2948
	rQIF-MRr	AR(1)	*β*_0_	3.0503	0.2901	0.2944	3.0339	0.3014	0.3033
			*β*_1_	-4.9251	0.1698	0.1856	-4.9098	0.1679	0.1907
			*ψ*_1_	1.5355	0.3352	0.3371	1.5332	0.3613	0.3628
			*ψ*_2_	0.2267	0.2618	0.2908	0.2281	0.2720	0.3007
			*ψ*_3_	5.6058	0.1846	0.2127	5.6060	0.1979	0.2245
		Exchangeable	*β*_0_	3.0282	0.3621	0.3632	3.0463	0.3371	0.3402
			*β*_1_	-4.8960	0.1728	0.2016	-4.9026	0.1741	0.1995
			*ψ*_1_	1.5156	0.3482	0.3485	1.5032	0.3687	0.3687
			*ψ*_2_	0.2070	0.2559	0.2774	0.2198	0.2571	0.2836
			*ψ*_3_	5.6102	0.2042	0.2320	5.5970	0.1978	0.2203
		Unspecified	*β*_0_	3.0027	0.4507	0.4507	2.9941	0.3957	0.3957
			*β*_1_	-4.9160	0.2012	0.2180	-4.9011	0.1739	0.2000
			*ψ*_1_	1.5389	0.4213	0.4231	1.5199	0.4691	0.4696
			*ψ*_2_	0.2321	0.2768	0.3067	0.2415	0.2448	0.2827
			*ψ*_3_	5.6229	0.2122	0.2452	5.6142	0.1927	0.2240

When the correlation is medium (*ρ* = 0.5), and high (*ρ* = 0.95) in Tables 3 and 4 respectively, estimation using misspecified working correlation structures still gives unbiased and efficient estimates with small SE and RMSE. Although the true model (i.e. AR(1)) gives slightly better estimates, but the difference is not far. This is one of the advantage of using the QIF in estimation, where the parameter estimates is efficient even with the misspecified working correlation structure [24, 34].

## Application to Warfarin data

For application, we use the data from Warfarin-treated patients who at risk of thrombosis [2]. The data consists of 303 patients with 14 clinic visits. The states, *S*_*j*_, is defined as the difference between the International Normalized Ratio (INR) at visit *j* and the INR within the target range. Meanwhile, *A*_*j*_ is defined as the dose given to patients at each visit *j*. Suppose the INR is used to measure blood clotting speed, and a positive *S*_*j*_ indicates that the clotting time was too long and that the dose *A*_*j*_ should be reduced, and vice versa. The goal of the treatment is to make sure the INR is within the target range.

From the 14 clinic visits, only 9 visits were considered, where the first 4 visits were treated as a stabilization period, and the last visits had no contribution to the outcome. At each visit *j*, the response *Y*_*j*_ is measured for *j* = 1, 2, …, *K* with *K* = 9. Hence, the mean response for *Y*_*j*_ conditional on $\left({\bar{S}}_{j},{\bar{A}}_{j}\right)$ as in Eq (1). The mixture model for *S*_*j*_ is used to obtain the state residuals, *Z*_*j*_. The model consists of a logistic component for *P*(*S*_*j*_ = 0) and linear component for |*S*_*j*_| given (*S*_*j*_ ≠ 0).

The regret function is modeled as in Eq (9). The first step will be estimating the residual of the state function, *Z*_*j*_. Then, for each *i*, we estimate the *g*_*i*_(*β*, *ψ*) functions, the extended score matrix *g*_*n*_(*β*, *ψ*), and the partial derivatives $\partial h_{i}^{T}/\partial \left(\beta ,\psi \right)$. In estimation, the initial value for (*β*, *ψ*) = {8.00, 0.00, 2.00, 0.25, −5.00} given that (*β*, *ψ*) is unknown for this application [13]. The parameter estimates of $\left(\hat{\beta },\hat{\psi }\right)$ can be obtained as in Eqs 6 and 8 for QIF-MRr and rQIF-MRr respectively. Using a cross-validation technique, the optimal tuning parameter λ = 0.1375436. 100 bootstrap resamplings were performed to test the consistency and efficiency of the estimation using AR(1), exchangeable, and unspecified working correlation structures.

Table 5 shows the results of parameter estimations for Warfarin data using the QIF-MRr and rQIF-MRr with three different types of working correlation structures. In comparison to AR(1) with an unspecified working correlation structure, estimation using rQIF-MRr with an exchangeable working correlation structure is more efficient with a smaller SE. Estimation using the rQIf-MRr with the AR(1) working correlation structure produces better results than the QIF-MRr. Meanwhile, the findings for both methods are almost similar when we estimate the parameters using an exchangeable and unspecified working correlation structure.

**Table 5:** Parameter estimation of Warfarin data with AR(1), exchangeable, and unspecified working correlation structures for QIF-MRr and rQIF-MRr.

Method		$\hat{\beta_{1}}$	$\hat{\beta_{2}}$	$\hat{\psi_{1}}$	$\hat{\psi_{2}}$	$\hat{\psi_{3}}$
QIF-MRr_*AR*(1)_	Mean	10.5870	1.6317	2.5877	0.0720	-8.2011
	SE	2.9063	2.6250	1.8113	1.3275	4.9005
QIF-MRr_*Exchangeable*_	Mean	6.5295	-2.2246	0.6014	-0.0254	-2.5608
	SE	0.3169	0.1561	0.1246	0.0418	0.4755
QIF-MRr_*Unspecified*_	Mean	6.9895	-2.0406	1.0450	-0.0452	-3.4159
	SE	0.1733	0.2822	0.1606	0.0810	0.3609
rQIF-MRr_*AR*(1)_	Mean	6.7960	-2.3492	1.1033	-0.1296	-3.2082
	SE	0.4594	0.4512	0.5022	0.1987	0.4037
rQIF-MRr_*Exchangeable*_	Mean	6.5844	-2.0634	0.6057	-0.0566	-2.8881
	SE	0.2497	0.1261	0.1742	0.1711	0.2019
rQIF-MRr_*Unspecified*_	Mean	6.9572	-2.0550	1.0379	-0.0383	-3.4174
	SE	0.2037	0.2756	0.1662	0.0585	0.3791

## Conclusions

The rQIF-MRr method was proposed in this paper with the goal of improving estimation in ODTR. There is huge potential to explore rQIF-MRr in personalized medicine, particularly in estimating ODTR. The study of ODTR is a branch of personalized medicine and it is a promising and developing field.

The simulation studies show that the parameter estimation using rQIF-MRr is unbiased and efficient even with the misspecified working correlation structure. In the simulation analysis, the proposed rQIF-MRr method performed well for small and large sample sizes at any correlation level. In comparison to the QIF-MRr, parameter estimation using the rQIF-MRr gives an efficient estimate with a minimal standard error when applied to Warfarin data.

Comparisons of different methods for determining the optimal tuning parameter λ, such as generalized cross-validation technique [35, 36], are in our best interest. Genetic algorithm [37] and particle swarm optimization [38] are two other methods.

When working with participants or patients with a defined time study in ODTR, there may be dropouts during data collection. It’s possible that this will result in either missing or survival data. The proposed method is incompatible with this type of data. Improvisation is required to deal with missing and survival data.

## Supporting information

S1 Dataset (DAT)
